# G Protein-Coupled Estrogen Receptor-1 Is Involved in the Protective Effect of Protocatechuic Aldehyde against Endothelial Dysfunction

**DOI:** 10.1371/journal.pone.0113242

**Published:** 2014-11-20

**Authors:** Byung Soo Kong, Yoon Hee Cho, Eun Jig Lee

**Affiliations:** Institute of Endocrine Research and Brain Korea 21 Project for Medical Science, Endocrinology, Yonsei University, College of Medicine, Seoul, Korea; Faculty of Medicine & Health Sciences, United Arab Emirates

## Abstract

Protocatechuic aldehyde (PCA), a phenolic aldehyde, has therapeutic potency against atherosclerosis. Although PCA is known to inhibit the migration and proliferation of vascular smooth muscle cells and intravascular thrombosis, the underlying mechanism remains unclear. In this study, we investigated the protective effect of PCA on endothelial cells and injured vessels *in vivo* in association with G protein-coupled estrogen receptor-1 (GPER-1). With PCA treatment, cAMP production was increased in HUVECs, while GPER-1 expression was increased in both HUVECs and a rat aortic explant. PCA and G1, a GPER-1 agonist, reduced H_2_O_2_ stimulated ROS production in HUVECs, whereas, G15, a GPER-1 antagonist, increased ROS production further. These elevations were inhibited by co-treatment with PCA or G1. TNFα stimulated the expression of inflammatory markers (VCAM-1, ICAM-1 and CD40), phospho-NF-κB, phospho-p38 and HIF-1α; however, co-treatment with PCA or G1 down-regulated this expression significantly. Likewise, increased expression of inflammatory markers by treatment with G15 was inhibited by co-treatment with PCA. In re-endothelization, aortic ring sprouting and neointima formation assay, rat aortas treated with PCA or G1 showed accelerated re-endothelization of the endothelium and reduced sprouting and neointima formation. However, aortas from G15-treated rats showed decelerated re-endothelization and increased sprouting and neointima formation. The effects of G15 were restored by co-treatment with PCA or G1. Also, in the endothelia of these aortas, PCA and G1 increased CD31 and GPER-1 and decreased VCAM-1 and CD40 expression. In contrast, the opposite effect was observed in G15-treated endothelium. These results suggest that GPER-1 might mediate the protective effect of PCA on the endothelium.

## Introduction

Endothelial dysfunction is an imbalance between vasodilating and vasoconstricting substances produced by the endothelium leading to a proinflammatory state and prothrombic properties. Endothelial dysfunction is an important early event in the pathogenesis of atherosclerosis. Mechanisms that participate in endothelial dysfunction include reduced nitric oxide generation, oxidative excess, and upregulation of adhesion molecules [7], [8]. Previous studies on atherosclerosis have provided some information on this topic. For example, increased expression of proteins such as VCAM-1, ICAM-1, E-selectin, CD40, lectin-like oxidized LDL receptor-1 (LOX-1) [9], production of matrix metalloproteinases (MMPs) [10] and reactive oxygen species (ROS), and decreased secretion levels of NO [11] contribute to both initiation and progression of atherosclerosis.

Estrogen has protective effects against cardiovascular diseases, and its receptors ER α and ER β have been shown to mediate anti-atherogenic effects. Recently, a third membrane-bound ER has emerged, G protein-coupled estrogen receptor-1 (GPER-1), that has beneficial effects on the cardiovascular system. GPER-1 is a seven transmembrane-domain G protein-coupled receptor (GPCR) that binds to 17β-estradiol (E2) with high affinity and mediates estrogenic signals [12]. GPER-1 is widely expressed in human tissues, including the cardiovascular system [13], [14]. It was recently found that selective activation of GPER-1 potently inhibits the growth of human vascular smooth muscle cells [15]. To find the role of GPER-1 on endothelial protection, G1 (GPER-1 agonist) and G15 (GPER-1 antagonist) have been evaluated. These pharmacological agents are currently used most frequently as tools for investigating the role of GPER-1 in various systems [12]. In this study, both agents were used to modulate GPER-1 *in vitro* and *in vivo* to investigate the protective role protocatechuic aldehyde (PCA) has in endothelial dysfunction through GPER-1.

Protocatechuic aldehyde (PCA) is a phenolic aldehyde found in the aqueous extract of *Salvia Miltiorrhiza* that has recently been reported for its anti-oxidative effects. It was recently reported that PCA reduces myocardial infarct size and the activities of creatine kinase-MB and cardiac troponin in serum [16]. Also, it can inhibit migration and proliferation of vascular smooth muscle cells and intravascular thrombosis [17]. However, the underlying mechanism of PCA on reducing inflammation and its effects on endothelial dysfunction remains to be determined. In this study, we investigated the protective effect of PCA on endothelial cells and injured vessels *in vivo* in association with GPER-1.

## Materials and Methods

### Reagents, Antibodies and assay kits

**Figure 1:**
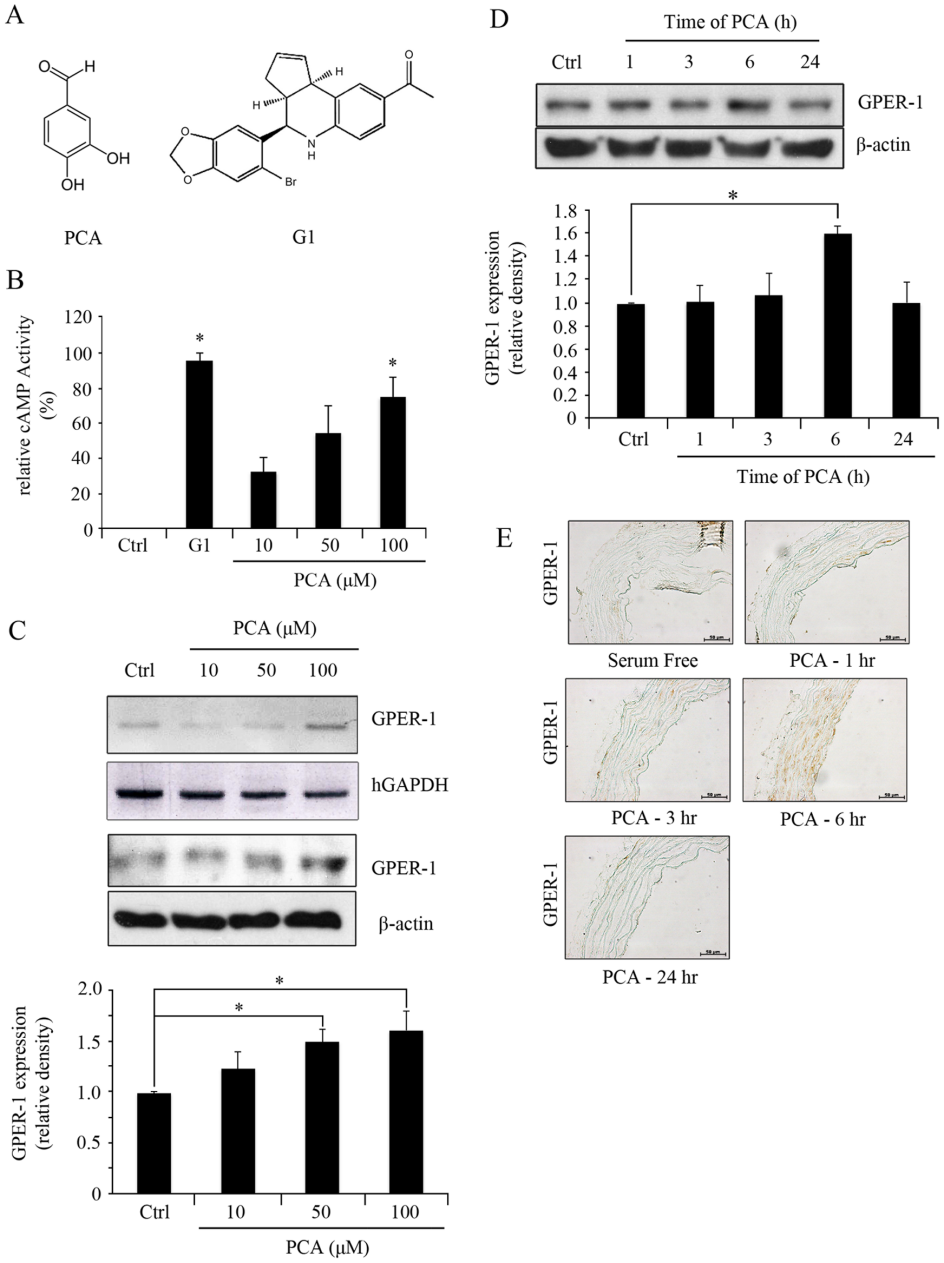
PCA induces expression of GPER-1. (**A**) The chemical structure of PCA and G1. (**B**) HUVECs were treated with G1 (3.0 µM) and PCA (10, 50, or 100 µM) for 1 hr to measure the levels of cAMP activity. (**C**) HUVECs were treated with PCA (100 µM) over 1, 3, 6, 24 hrs. (**D**) HUVECs were treated with PCA (10, 50, or 100 µM) for 6 hrs. Blots are representative of 3 independent experiments. Densitometric analyses are presented as the relative ratio of GPER-1 to β-actin. Data are presented as mean±SEM; * P<0.05. (**E**) Aortic segments were harvested from Sprague Dawley rats and cultured *ex vivo* in serum-free media with PCA (100 µM) over 1, 3, 6, 24 hrs. Segments were than fixed for immunohistochemistry of GPER-1 using 4% paraformaldehyde.

Protocatechuic aldehyde (PCA) and GPER-1 agonist, G1 (Figure 1 A) were purchased from Sigma Aldrich (St. Louise, MO, USA). GPER-1 antagonist, G15 was purchased from Calbiochem (San Diego, CA, USA). TNF-α and rat PDGF-BB were purchased from R&D Systems (Minneapolis, MN, USA).

Antibodies for western blot analysis against GPER-1 (sc-134576), VCAM-1 (sc-8304), ICAM-1 (sc-7891), CD31 (sc-1506), CD40 (sc-975) were purchased from Santa Cruz (Delaware, CA, USA). phospho-MAPK (#9106S), NFκB (#4767), phospho-NFκB (#3033S), and HIF-1α (#3716S) were purchased from Cell Signaling Technology (Danvers, MA, USA).

For ROS activity assay, CM-H2DCFDA kit (#C6827) was bought from Invitrogen (Carlsbad, CA, USA). For sprout ring assay, Cultrex BME matrigel (3431-005-01) was bought from Trevigen (Gaithersburg, MD, USA). MTS assay kit (G3580) was purchased from Promega (Madison, WI, USA). Cyclic AMP XP Assay kit (#4339) was brought from Cell signaling (Danvers, MA, USA)

### Ethics Statement

All animal procedures were reviewed and approved by the Institutional Animal Care and Use Committee (IACUC) of Yonsei University Health System (approval number: 2010-0268) and were performed in strict accordance with the Association for Assessment and Accreditation of Laboratory Animal Care (AAALAC).

### Cell Culture

Primary HUVECs were obtained from GIBCO (C-003-5C). HUVECs were cultured in EBM-2 (CC-3129) supplemented with serum kit (CC-4176) purchased from Lonza (Walkersville, MD, USA). Cells were maintained at 37°C humidified atmosphere of 5% CO_2_ incubator in collagen-coated dish supplied from Sigma Aldrich (C9791). The medium was changed every 2 days. For the experiments, HUVECs passage 3∼8 were used after 2 hours of serum depletion.

### Western Blotting

Western blot analysis was performed using antibodies against GPER-1, VCAM-1, ICAM-1, CD31, CD40, phospho-MAPK, phospho-NFκB, and HIF-1α. Cells were lysed in buffer containing 10 mM Tris, 400 mM NaCl, 1 mM EDTA, 0.1% NP-40. Protein concentration of the cell was determined by the Bradford method. Equal amounts of the whole protein extract were resolved on 10% SDS gels followed by semi-dry Western blotting. Subsequently, membranes were blocked with 5% BSA in TBST (50 mM Tris-HCl, pH 7.5 and 150 mM NaCl containing 0.05% Tween 20). Blots were probed overnight with the dilutions of antibodies listed above in 5% BSA containing TBST (10 mM Tris pH 8.0, 150 mM NaCl, 0.05% Tween-20). After incubation with a secondary anti-rabbit antibody coupled to horseradish peroxidase immunocomplexes were visualized by enhanced chemi-luminescence.

### Animal model – Common Carotid Balloon injury

Male Sprague Dawley rats (SD rats, ORIENT-Charles River Technology, Seoul, Korea) weighing 200∼225 were housed in a temperature-controlled environment (24∼26°C) with a 12/12-hours reversed light and dark cycle, in a plastic cage with soft bedding, and given with a free access to tap water and standard laboratory chow.

7 weeks old rats were randomly divided into seven groups and they were daily fed with the following amounts: Group 1 – remained intact; Group 2 – Balloon injury; Group 3 – PCA (100 mg/kg) with balloon injury; Group 4 – G1 (3.0 mg/kg) with balloon injury; Group 5 – G15 (3.0 mg/kg) with balloon injury; Group 6 – G15 (3.0 mg/kg) and PCA (100 mg/kg) with balloon injury; Group 7 – G15 (3.0 mg/kg) and G1 (3.0 mg/kg) with balloon injury.

For operative procedures, SD Rats were anesthetized with 5% isoflurane in a mixture of 70% N_2_O and 30% O_2_ and it was maintained with 2% isoflurane. Also during this procedures, body temperatures were constantly checked with a rectal probe to maintain at 37.0±0.2°C using a homeothermic blanket control unit and a heating pad (Harvard Appratus, Holliston, MA, USA). After 2 weeks of intraperitoneal administration, rats' left common carotid arteries were isolated and a 2F Forgarty catheter (Edwards Lifesciences, Irvine, CA, USA) was introduced through the external carotid arteriotomy incision, advanced to the aortic arch, inflated to produce moderate resistance, and gradually withdrawn 7 times as previously reported [1].

For Neointimal Formation experiment, animals were injected with each substance for 4 weeks more after 2 days of recovery and then sacrificed.

For Re-endothelization assay, animals were injected with each substance for 1week more after 2 days of recovery and then injected with Evans Blue through tail vein. They were left injected for 30 minutes and then sacrificed to obtain the common carotid artery for evaluation. During these procedures, every effort was made to minimize animal usage and their suffering.

### Measurement of ROS (Reactive Oxygen Species) in HUVECs

Levels of cellular reactive oxygen species were measured using the fluorescent probe 5-(and-6)-chloromethyl-2′, 7′-difluorodihydrofluorescein diacetate (CM-H2DFFDA). To prepare for the ROS assay, HUVECs cultured in EBM-2 supplemented with serum kit were changed into serum free media with 0.1% serum. Sample is treated for 24 hours and then, 1 hour of H_2_O_2_ 100 uM is added to each dish. After that, ROS assay procedures were followed as it was described from other studies [2]. Samples were analyzed with FACS Calibur (BD Biosciences, Franklin Lakes, NJ, USA).

### cAMP activity assay in HUVECs

HUVECs were seeded on 96 well plates in EBM-2 media with serum kit. After a day of incubation, serum media were replaced with serum free EBM-2 media with 0.1% FBS serum. After 2 hours of incubation, appropriate substances (Control, PDGF, PCA, G1, G15, G15+PCA, G15+G1) were added and incubated for 1 hr. Then steps were followed as it is stated on the manufacturer's manual from Cell Signaling (Cell Signaling, Danvers, MA, USA).

### *Ex vivo* Sprague Dawley Rat's thoracic aorta culture

Male Sprague Dawley Rats (100 g) were housed in a controlled environment as previously described. After one week of stabilization, under anesthesia, rats were sacrificed and thoracic aortas were obtained. Thoracic aortas were chopped into several pieces and placed on Cell Culture Insert (PICM03050) purchased from Millicell (Billerica, MA, USA) in 6-well plate.

Aortas were maintained in EBM-2 media with serum kit for one day to make them adapt to the experiment condition. Before the treatment of PCA, EBM-2 media with serum kit were removed and replaced with EBM-2 serum free media. After 2 hours of serum depletion, PCA were added in time dependent manner. After the treatment, aortas were stored in 4% paraformaldehyde for fixation in 4°C condition.

### Sprout Ring assay

This method was used with some modifications by following previous work originally reported for mice aorta [3]. Under anesthesia, thoracic aortas were removed from rat of 100 grams and transferred to a 50 ml conical tube containing ice-cold serum-free EBM-2 medium. The peri-aortic fibro-adipose tissue was carefully removed with fine micro-dissecting forceps not to damage the aortic wall. Half a millimeter long aortic rings (approximately 25 per aorta) were sectioned. Ring-shaped explants of rat aorta were then embedded in BME Matrigel on Coverglass Bottom dish. EBM-2 serum media was added into the dish and left to be incubated for 3 days. 3 days later, PCA, G1, G15, G15 and PCA, and G15 and G1 were added. The cultures were kept at 37°C in a humidified environment for a week and examined every day with an Olympus microscope at appropriate magnification.

### Vascular Histology and Immunohistochemical procedures

Rat arteries were perfused with saline, removed, and fixed in 4% paraformaldehyde for 24 hours in 4°C. Then, they were embedded in paraffin and prepared in 4 µm cross sections. Rat aortas were stained with hematoxylin and eosin using standard protocol [4], [5]. For immunohistochemical analyses, sections were deparaffinized in xylene, rehydrated in graded ethanol solutions and washed with distilled water as described from other studies [6]. Sections were blocked with 5% goat serum (005-000-121, Jackson ImmunoResearch, West Grove, PA, USA) in antibody diluent (S2022, Dako, Glostrup, Denmark) for 30 minutes and incubated overnight at 4°C with following antibodies: GPER-1, CD40, CD31 and VCAM-1 (1∶150). After washing three times in TBS-T, slides were incubated for 1 hr with a biotinylated secondary antibody (Vector Laboratories, Burlingame, CA, USA). After rinsing three times in TBS-T, RTU horseradish peroxidase streptavidin (SA5704, Vector Laboratories) was applied and the slides are incubated for 10 minutes. For color development, 3,3′-diaminobenzidine (DAB, D5637, Sigma) was used.

### MTS assay

VSMCs grown on 100 mm are trypsinized and seeded on 96 well plates. After 24 hours of incubation in DMEM serum media, serum media are replaced with serum free DMEM media. After 24 hours of serum depletion, appropriate drugs (Control, PDGF, PCA, G1, G15, G15 and PCA, G15 and G1) are added in serum free DMEM media to each group. For Control and PDGF group (10 ng/ml), no substances are added. After 24 hours of drug treatment, media are removed and replaced with PDGF added drug-containing DMEM media except for Control group. After 24 hours later, steps are followed as it is stated on the manufacturer's manual from Promega.

### Quantification of *in vitro* results, sprout length and neointima size of Rat arteries

To perform image analysis, all images were taken under the same observation condition (light, contrast, magnification). Image analysis was performed by using Scion Image software and results were expressed as mean±SEM. Differences between groups for both in *vivo* and *vitro* results were evaluated using SPSS 18.0 software. Also graphs for Balloon injury, reendothelization assay and sprout ring assay were done by MedCalc software program.

For morphologic analysis of neointimal formation, six round cross-sections (4 µm thickness) were cut from the approximate middle of the artery. The intimal and medial cross-sectional areas of the carotid arteries were measured, and intima/media ratios were calculated. Antibody against GPER-1 was used for immunohistochemistry.

## Results

### PCA increases GPER-1 expression in HUVECs and Sprague Dawley rat aortas

To assess whether the effect of PCA on human umbilical vein endothelial cells (HUVECs) is mediated through GPER-1, cAMP levels and GPER-1 expression were measured after PCA treatment. PCA increased cAMP levels in a concentration-dependent manner. Significant elevation of cAMP levels was observed at 100 µM of PCA. Comparison of elevated cAMP levels between G1 (3.0 µM) and PCA stimulation showed that PCA was less potent than G1. GPER-1 expression was increased by PCA treatment in a concentration dependent manner with the highest expression after 6 hrs of incubation compared to that of the control (1.61 fold, *P*<0.05) (Figure 1C, D). In the *ex vivo* culture of rat aortic explants, 100 µM of PCA increased GPER-1 expression after 6 hrs of treatment. (Figure 1E).

### PCA decreases ROS production in HUVECs

**Figure 2:**
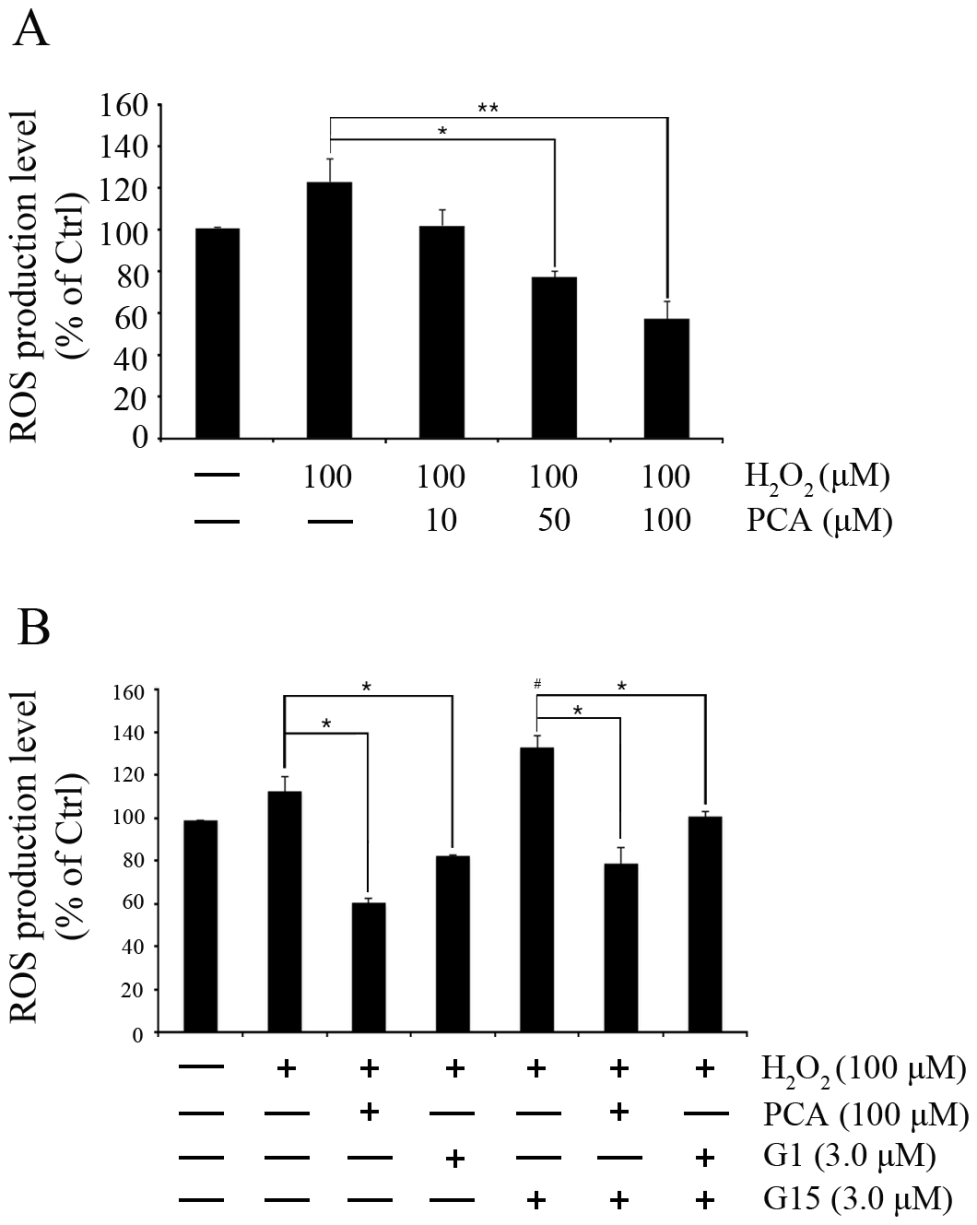
Protocatechuic aldehyde decreases ROS production. (**A**) HUVECs were pretreated for 24 hrs with various concentrations (10, 50, 100 µM) of PCA. (**B**) HUVECs were pretreated for 24 hrs with PCA (100 µM) and G1 (3.0 µM) and for 6 hrs with G15 (3.0 µM). Then, (**A, B**) HUVECs were treated with H_2_O_2_ (100 µM/ml) for 1 hr followed by measurement of ROS levels. (**A, B**) Both experiments are representative of 3 independent experiments. Data are presented as mean±SEM; * P<0.05, ** P<0.005, # indicates P<0.05 compared to the control group.

PCA reduced H_2_O_2_ stimulated ROS production in HUVECs in a concentration-dependent manner. Treatment with 50 or 100 µM of PCA significantly decreased ROS production (77.31±2.56%, *P*<0.05 and 57.18±8.33%, *P*<0.01) stimulated by 100 µM H_2_O_2_ for 1 hr (Figure 2A). Comparison between G1 (3.0 µM) and PCA (100 µM) showed similar decreases in ROS production. Whereas, the GPER-1 antagonist G15 (3.0 µM) increased ROS production further to 132.47±5.49% (*P*<0.05). This elevation in ROS production was decreased to 78.33±7.73% (*P*<0.05) and 100.25±2.41% (*P*<0.05) by co-treatment with PCA or G1, respectively (Figure 2B).

### PCA inhibits inflammatory signaling

**Figure 3:**
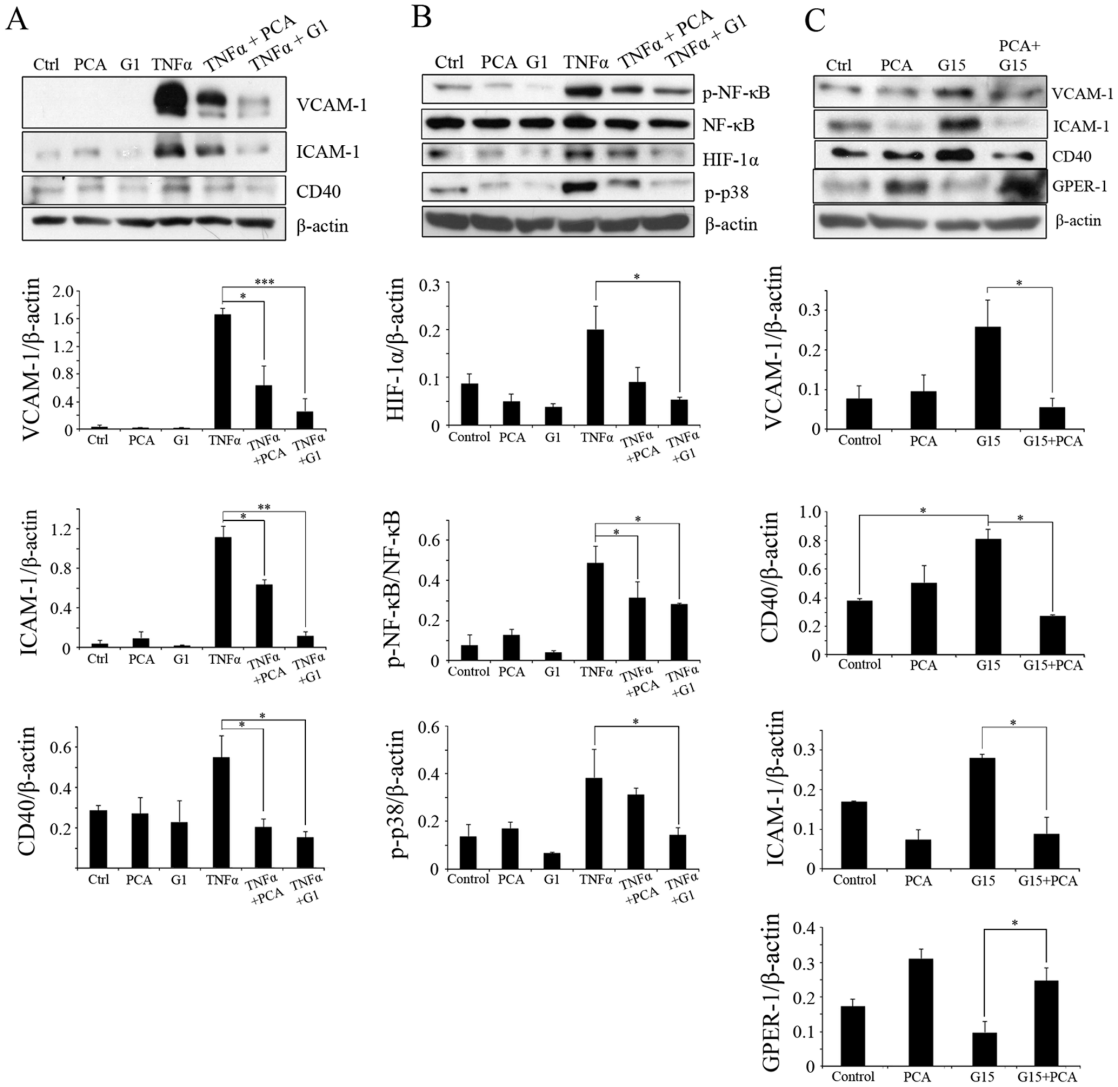
Human umbilical vein endothelial cells (HUVECs) treated with tumor necrosis factor alpha (TNFα) and G15 induces inflammation. (A, B) HUVECs were pretreated with PCA (100 µM) and G1 (3.0 µM) for 24 hrs. (A) treatment with TNFα (10 ng/ml) for 6 hrs. (B) treatment with TNFα (10 ng/ml) for 1 hr. (C) HUVECs were pre-treated with PCA (100 µM) for 24 hrs and then treated with G15 (3.0 µM) for 6 hrs. Blots are representative of 3 independent experiments. (A, B, C) Densitometric analyses are presented as the relative ratio of ICAM-1, VCAM-1, CD40, HIF-1α, GPER-1 or phospho-p38 to β-actin. (B) Densitometric analyses for phospho-NF-κB are presented as the relative ratio to NF-κB. Data are presented as mean±SEM; * P<0.05, ** P<0.005, *** P<0.001.

To evaluate whether PCA could down-regulate the inflammation in HUVECs caused by TNFα treatment, HUVECs were pre-treated with PCA (100 µM) or G1 (3.0 µM) for 24 hours [17]. Then, 10 ng/ml TNFα was added for 6 hours to provoke inflammation. As shown in Figure 3A, TNFα stimulated the expression of VCAM-1, ICAM-1 and CD40; however, PCA down-regulated these expression levels significantly. G1 also down-regulated their expression in a similar manner (Figure 3A).

To investigate inhibition of the NF-κB signaling pathway by treatment with PCA or G1, endothelial cells were pre-treated with PCA (100 µM) or G1 (3.0 µM) in the presence or absence of TNFα (10 ng/ml, 1 hr). As shown in Figure 3B, TNFα increased the expression of phospho-NF-κB, phospho-p38 and HIF-1α. This elevated expression was down-regulated significantly by PCA and G1 (Figure 3B).

We also investigated whether G15, an antagonist of GPER-1, affects the expression of inflammatory markers in HUVECs and if PCA could act as a GPER-1 activator to block the activity of G15. As shown in Figure 3C, G15 showed GPER-1 inhibition activity by decreasing the expression level of GPER-1. Also, G15 increased VCAM-1, ICAM-1 and CD40 compared to the control. However, PCA treatment increased GPER-1 and inhibited inflammation markers dramatically.

### PCA inhibits angiogenesis

**Figure 4:**
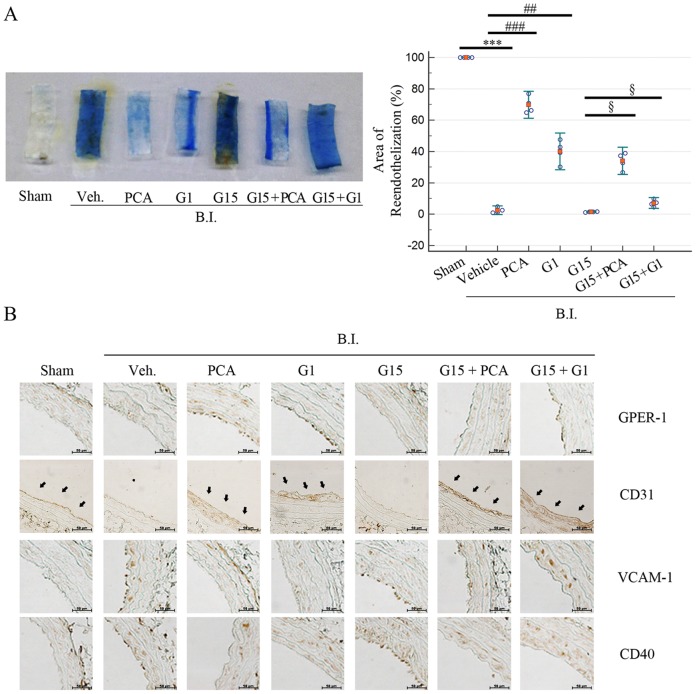
Protocatechuic aldehyde exhibits endothelial protection. (**A**) Re-endothelization of a Sprague Dawley rat carotid artery determined by Evans Blue staining (de-endothelized areas are stained with blue). Ratios of surface covered by endothelium to the total area in sham, vehicle, and injured (Inj) groups treated with different substances as follows: PCA, G1, G15, G15+PCA, G15+G1 (n = 4 each). (**B**) Cross sections of rat common carotid arteries stained with CD31 antibody 3 days after injury. Also, targets related to inflammatory markers were observed including VCAM-1, CD40 and GPER-1. Data are presented as mean±SEM; *** indicates P<0.001 compared to the sham group. ## and ### indicate P<0.005 and P<0.001, respectively, compared to the vehicle group. § indicates P<0.05 compared to the G15+ Inj. group.

To investigate the effect of PCA on angiogenesis, the Sprout Ring assay was performed. As shown in Figure 4A, treatment with PCA (3.74±1.89%, *P*<0.001) or G1 (5.49±2.83%, *P*<0.001) significantly prevented the length of sprouting compared to the serum only group (100.0%). Whereas, the addition of G15 (223.9165±32.96%, *P*<0.01) dramatically increased the sprouting of VSMCs. Co-treatment with PCA or G1 inhibited sprouting dramatically in Figure S1.

### Protocatechuic aldehyde accelerates re-endothelization in balloon-injured arteries

To investigate whether PCA also protects the endothelium through GPER-1 under *in vivo* conditions, endothelial recovery was evaluated after balloon denudation of the carotid artery in rats [18].

**Figure 5:**
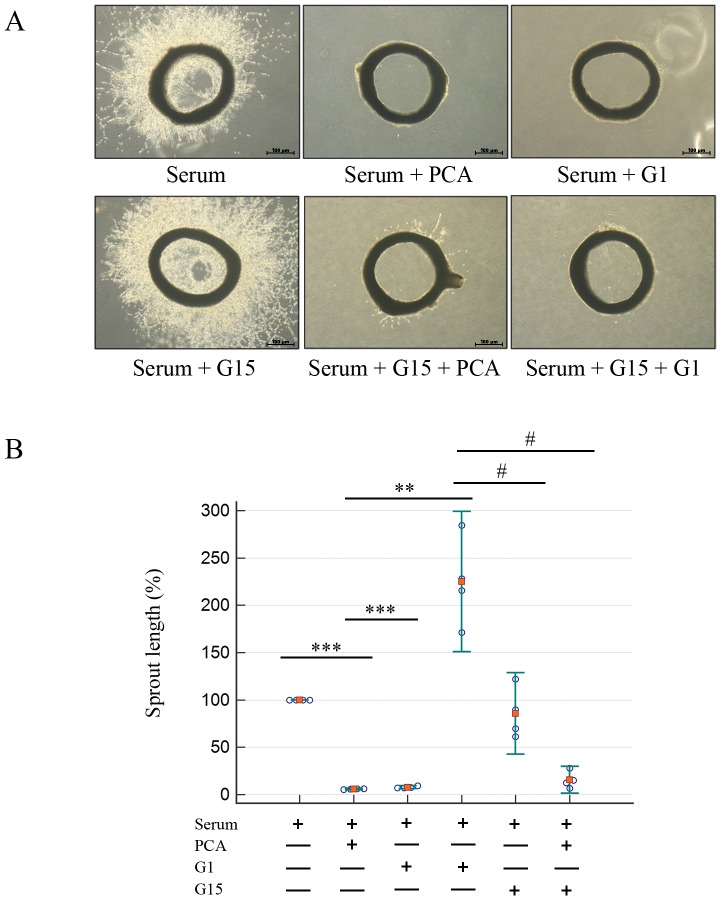
PCA inhibits angiogenesis. (**A**) Rat aortas were obtained from Sprague Dawley rats weighing 100 grams. Rat aorta segments placed on Matrigel were stimulated with FBS (10%) for 3 days. Then, the segments were treated with each substance for 48 hrs in media supplemented with FBS (10%). Sprout lengths were measured by using Scion Image software. (**A, B**) Values represent the means ± SEMs of 3 experiments. *** indicates P<0.001 compared to the control group. ## and ### indicate P<0.005 and P<0.001, respectively, compared to the PDGF group. §§§ indicates P<0.001 compared to the G15 group. Production will need this reference to link the reader to the figure.

Sprague Dawley rats were pre-treated with the substances for 2 weeks by intraperitoneal injection and then balloon injury of the common carotid artery was performed. Rats were injected with the substances for another 3 days after the surgical procedure. Before the isolation of carotid arteries, rats were injected with Evans Blue through the tail vein and left for 30 min. As shown in Figure 5A, treatment with PCA or G1 accelerated re-endothelization in balloon-injured arterial segments. The re-endothelized area in the PCA- and G1-treated rats was 69.35±3.81% (*P*<0.001) and 40.21±5.21% (*P*<0.01), respectively, compared to the vehicle rats. The effect of PCA on re-endothelization was higher compared to that of G1. G15-treated rats did not show re-endothelization, similar to the vehicle treatment. Co-treatment of PCA on G15 treated aortas showed effective re-endothelization (42.77±13.17%, *P*<0.05), whereas co-treatment of G1 on G15 treated aortas showed partial effects (6.90±1.52%, *P*<0.05) (Figure 5A).

As shown in Figure 5B, immunostaining for CD31, an endothelial marker, showed that the PCA- and G1-treated groups had a clear line of CD31 along the endothelium of the artery. Whereas, the vehicle- or G15-treated groups showed no staining of CD31. This suggests that re-endothelization occurred in PCA- or G1-treated arteries. GPER-1 expression was increased in PCA- and G1-treated endothelium compared to vehicle-treated arteries. Treatment with G15 decreased expression of GPER-1. Co-treatment with G15 and PCA or G1 did not suppress GPER-1 completely, but decreased expression of VCAM-1 and CD40 was observed. The expression of VCAM-1 and CD40 was increased by G15 treatment; however, co-treatment with PCA or G1 decreased their expression (Figure 5B) in the endothelium.

### PCA inhibits neointima formation in balloon-injured common carotid arteries (CCAs)

We investigated the protective effect of PCA and G1 in a balloon injury model. Sprague Dawley rats (n = 7) were treated for 2 weeks with the substances before the surgical procedure [19]. After balloon injury of the CCA, rats were treated again with the substances for 4 weeks. After sacrifice, injured CCAs were isolated and paraffin embedded for H&E staining and immunohistochemistry for GPER-1.

**Figure 6:**
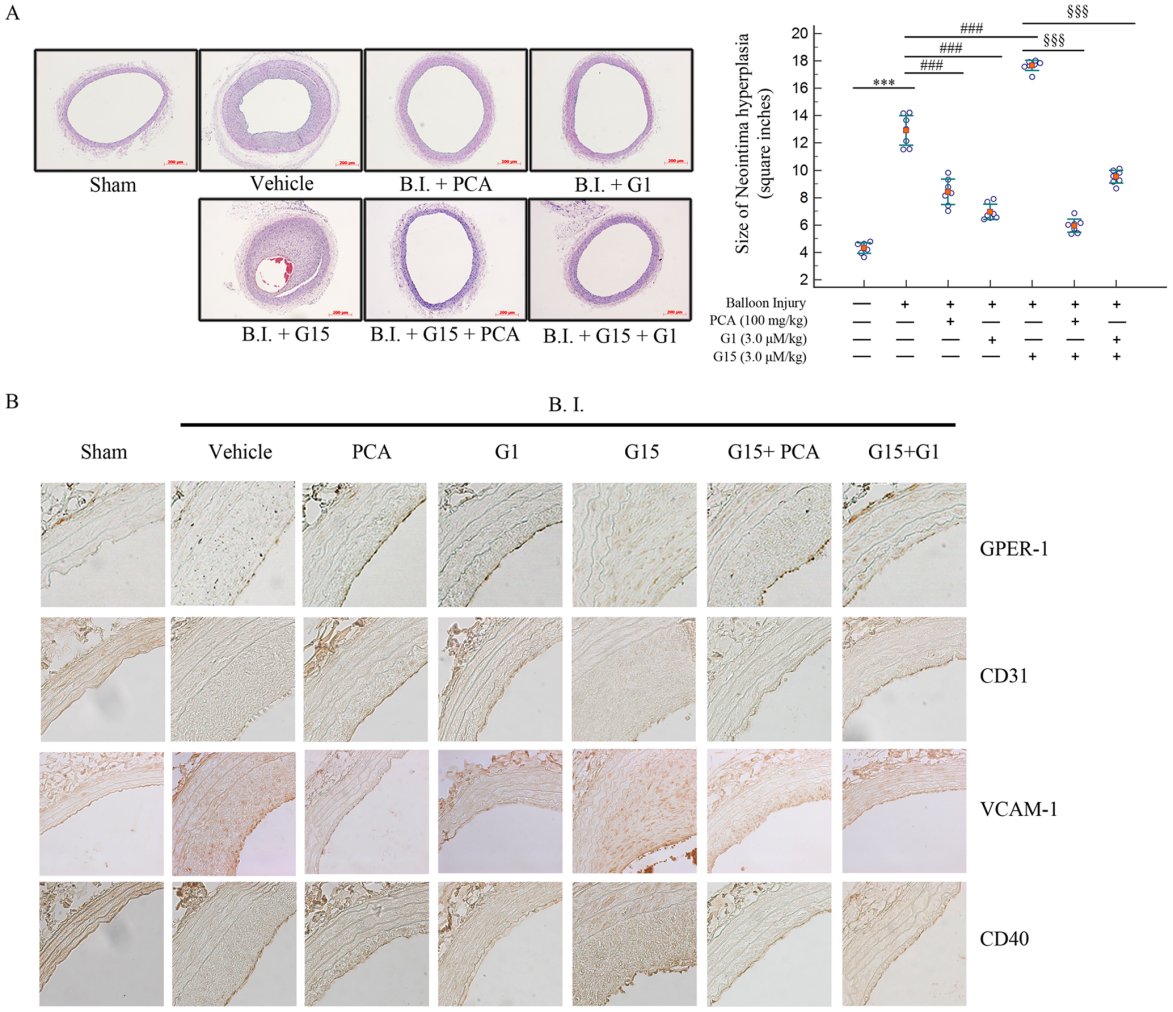
The effects of PCA, G1, G15, G15+PCA, and G15+G1 in CCA balloon-injured Sprague Dawley rats. (**A, B**) Seven-week-old Sprague Dawley rats (200 g) were treated with each substance by intraperitoneal injection for 2 weeks and then common carotid arteries from the rats were balloon injured. Substances were injected for another 4 weeks and then sacrificed for (**A**) H & E staining of common carotid arteries. Graph shows the percentage of neointima areas of Sprague Dawley rats from each group (n = 7). Measurements were made using Scion Image software. (**B**) immunohistochemistry of rat aortas shows GPER-1 expression in the linings of the aorta from the same tissues used for Figure 5A. Data are presented as mean±SEM; *** indicates P<0.001 compared to the sham group. ### indicates P<0.001 compared to the vehicle group. §§§ indicates P<0.001 compared to the G15 + Inj. group.

Figure 6A shows the representative images of each group treated with drugs along with bar graphs below. The results clearly show the effectiveness of PCA and G1 against neointima formation, whereas G15 increased formation. PCA and G1 attenuated neointimal hyperplasia from 14.00±0.95 inches^2^ to 9.65±0.76 inches^2^ (*P*<0.001) and 8.418±0.73 inches^2^ (*P*<0.001), respectively. However, in G15-treated rats, neointima formation dramatically accelerated to 17.16±1.06 inches^2^ (*P*<0.001). The effect of G15 was inhibited by co-treatment with PCA (6.06±0.39 inches^2^, *P*<0.001) or G1 (8.45±0.58 inches^2^, *P*<0.001).

Immunohistochemistry on Figure 6B shows that not only in early stages of atherosclerosis (Figure 4B) but also after the formation of neointima, the VCAM-1, CD40, GPER-1 and CD30 follow the similar pattern. This suggest that the treatments of G1 and PCA can effectively down-regulate the inflammatory markers (VCAM-1 and CD40) and up-regulate GPER-1 and CD31 to attenuate atherosclerosis for both short and long term. This data proposes PCA as an effective agent against atherosclerosis.

## Discussion

PCA has exhibited various therapeutic effects in different cell types such as vascular smooth muscle cells, cancer cells, and a cardiac animal model [17], [20], [21]. However, PCA has not been evaluated for its effect against endothelial dysfunction and has rarely been studied in HUVECs. Unlike PCA, GPER-1 has been consistently reported for its role in attenuating atherosclerosis because it regulates the activity of many vasoconstrictors and proliferation of vascular smooth muscle cells [12]. Thus, it is becoming increasingly difficult to ignore the importance of endothelial cells and GPER-1 in developing therapeutic agents against endothelial dysfunction and atherosclerosis.

The activation of GPER-1 by PCA protects endothelial cells in various ways from inflammation *in vitro*. One of them is the inhibition of ROS production, which is closely related to NF-κB. The reduction of ROS by PCA and GPER-1 agonist has shed light on the relationship between GPER-1 and NF-κB. Previous studies have pointed out the beneficial effects of GPER-1 on atherosclerosis, but they have failed to find connections with NF-κB [22]. However, we found that G1 and PCA could down-regulate the secondary molecules involved in NF-κB signaling. Such cytokine-mediated upregulation of NF-κB was shown to implicate HIF-1α [23], CD40 and other adhesion molecules (E-selectin, VCAM-1 and ICAM-1) [24], [25], which were effectively inhibited by PCA and GPER-1 agonist. We also showed that PCA and G1 down-regulated the phosphorylation of p38-mitogen activated protein kinase, which also has been recently reported from a previous study using ApoE^−/−^ mice [26]. Although other possible mechanisms have yet to be ruled out, our results strongly suggest a model in which PCA increases GPER-1 to down-regulate inflammatory molecules.

The activation of GPER-1 as a type of GPCR can result in beneficial effects against inflammation. Cyclic-AMP and Ca^2+^ are most frequently associated with GPER-1 signaling. GPER-1 couples to Gα_s_ resulting in cAMP production and Gα_i/o_ to partly yield cAMP [12]. PCA and G1 have been shown to increase the production of cAMP, but it remains unclear whether the GPCR α subunit of PCA can or cannot be activated. The activation of GPCR automatically activates regulatory molecules, including GRKs [27] and β-arrestin [28]. These molecules determine the role of GPER-1 once they are desensitized. It still remains unclear whether PCA or G1 can alter the expression of these molecules to change the fate of GPER-1.

PCA and G1 had inhibitory effects against angiogenesis in the Sprout Ring assay. In contrast, G15 treatment showed that it accelerated angiogenesis to a much greater extent than the serum only group. From the MTS assay (Figure S1), PDGF increased VSMC proliferation, which was decreased by PCA and G1. Treatment with G15 increased proliferation, but this angiogenic effect of G15 was inhibited by co-treatment with PCA or G1. These results expand the effects of PCA as an anti-angiogenic agent and show that PCA could interact with proteins other than GPER-1 since most GPCRs and their kinases are known to accelerate angiogenesis [29].

G15, an antagonist of GPER-1, has been recently discovered and its effects have not been evaluated in endothelial cells. Its involvement in blocking estradiol 17β-D-glucuronide-induced cAMP production [30] and upregulation of the angiotensin AT_1_ receptor have been reported [31]. Our *in vitro* results, showed that G15 acts as a pro-inflammatory agent to increase the expression of inflammatory markers such as VCAM-1, ICAM-1 and CD40. Also, the pro-inflammatory characteristics of G15 were observed for angiogenesis, neointimal formation and attenuation of re-endothelization. To verify whether PCA can act against the effects of G15, HUVECs were pre-treated with PCA before treatment with G15. The results showed that PCA effectively inhibited the pro-inflammatory effects of G15. Consistent with these results, the CCA of balloon-injured rats showed that PCA inhibited the pro-inflammatory effects of G15 by reducing neointimal hyperplasia. In accordance with the balloon injury results, the endothelia of rat aortas co-treated with PCA and G15 showed noticeable recovery from balloon injury.

To reveal the correlation between HUVECs and aortas, we performed immunohistochemistry for GPER-1, CD40, CD31 and VCAM-1. We have observed that rats co-treated with PCA and G1 had definite expression of CD31 and GPER-1, suggesting that GPER-1 affects endothelial cell survival. Also, the immunohistochemistry results for CD40 and VCAM-1 showed that PCA and G1 effectively inhibited both these molecules, similar to observations from the HUVEC experiment. In contrast, aortas from G15-treated rats did not show expression of either GPER-1 or CD31. Surprisingly, immunohistochemistry for VCAM-1 and CD40 was clearly observed in the vehicle and G15-treated groups. Furthermore, co-treatment with G15 and PCA or G1 in rats showed recovery of CD31 and GPER-1, indicating that PCA and G1 act through GPER-1 in a similar manner.

To further explain the various roles of PCA in the endothelium, studies on the structure of PCA are required. O′Brien et al [32] showed that aldehyde has therapeutic effects against some human diseases. Therefore, we have previously compared two different protocatechuic substances, aldehyde and its acid form, in order to reveal the structural function of this particular molecule. We showed that protocatechuic acid does not have the same therapeutic effects as the aldehyde form. This provided us with information on the structural importance of this molecule with respect to endothelial dysfunction. Further research will be required to elucidate the structural function of PCA for the development of new drugs against atherosclerosis.

GPER-1 and ring-structured aldehyde may be closely associated with each other. A recent study by Hamza et al [33] showed that hydroxyproline, which is one of the ring-structured aldehydes, activates GPER-1 through a different mechanism from estrogen. They also suggested that it stabilizes the active site of GPER-1 by forming a network of hydrogen bonds between the residues of GPER-1. PCA and 3,4-dihydroxybenzaldehyde share many structural features with hydroxyproline that need to be evaluated to confirm whether PCA acts in a similar manner as hydroxyproline. However, in the present study, we compared data on PCA and G1 and showed that PCA exhibited similar therapeutic effects as G1, thus demonstrating the likelihood that PCA can act like hydroxyproline.

Currently, it is difficult task to find the exact mode of activation by PCA. So, by comparing the effects of G1 and PCA in the presence of G15, it would clarify the function of PCA whether it can act like G1 or GPER-1 agonist in activating cAMP. The results from Figure S2A show that the treatment of PCA or G1 in the presence of G15 decreased cAMP in similar degree in comparison to the HUVECs treated with PCA or G1 without G15. This shows that PCA and G1 share the similar mechanism in interacting with GPER-1 for cAMP activation and that their activity cannot be fully inhibited by the treatment G15. This implicates that PCA can interact with GPER-1 like G1 agonist. Further researches are needed to find whether PCA and GPER-1 bind directly or not. To further verify the relationship between PCA and GPER-1 and G15's intervention in this relationship, we have treated G15 to HUVECs to see whether G15 can inhibit the cAMP activity of GPER-1. In Figure S2A shows that G15 has a mild inhibitory effect on cAMP activity. Also, the co-treatment of G15 with PCA or G1 has decreased activity level compared to PCA or G1 only treated group. This shows that G15, which is unknown for its capability on GPER-1 mechanism, does a slight inhibition on cAMP activity of GPER-1 in HUVECs. Also similar patterns were observed in Figure S2B as it mildly inhibits p-AMPK, which is well-known downstream protein of cAMP, increased by PCA and G1. This data suggests G15's inhibitory effect depend less on cAMP inhibition to cause inflammatory and oxidative effects. We believe that there are non-cAMP pathways that G15 can act on during GPER-1 activation. Further researches are needed to verify the G15's action on GPER-1 as an antagonist of GPER-1.

In summary, the present study provides the first evidence that PCA attenuates endothelial dysfunction and atherosclerosis *in vitro* and *in vivo* through activation of GPER-1. These findings indicate that PCA is an important candidate compound for the treatment of atherosclerosis.

## Supporting Information

Figure S1 **PCA's inhibition on VSMC cell proliferation.** Media of VSMCs in 96 well were changed to serum free media for 24 hrs. Then, PCA (100 µM), G1 (3.0 µM) and G15 (3.0 µM) were treated for 24 hrs. After that, PDGF (10 ng/ml) was treated for 24 hrs. Graphs are representative of 3 independent experiments. *** indicates P<0.001 compared to the sham group. ##, ### indicates P<0.005, P<0.001, respectively to PDGF group. $$$ indicates P<0.0001 compared to PDGF+G15 group. Production will need this reference to link the reader to the figure.
(TIF)

Figure S2 **PCA and G15 effects on GPER-1 related mechanism.** (**A**) HUVECs in 96 well were treated with adequate substances (PCA, G1, G15, G15+PCA, G15+G1) for 1 hr after 2 hrs of serum depletion. Graphs are representative of 3 independent experiments. * indicates P<0.05 compared to the control and G15 treated group. (**B**) HUVECs were pretreated with adequate substances for 24 hrs (PCA, 100 µM; G1, 3.0 µM). Then G15 (3.0 µM) were added for 6 hrs for p-AMPK and AMPK. Blots are representative of 3 independent experiments. * indicates P<0.05 compared to the control group. # indicates P<0.05 compared to the G15 group.
(TIF)
