# Supplementary figures and images for: G Protein-Coupled Estrogen Receptor-1 Is Involved in the Protective Effect of Protocatechuic Aldehyde against Endothelial Dysfunction
Source: PLoS One. 2014 Nov 20;9(11):e113242. doi: 10.1371/journal.pone.0113242 (PMC4239058; doi:10.1371/journal.pone.0113242)

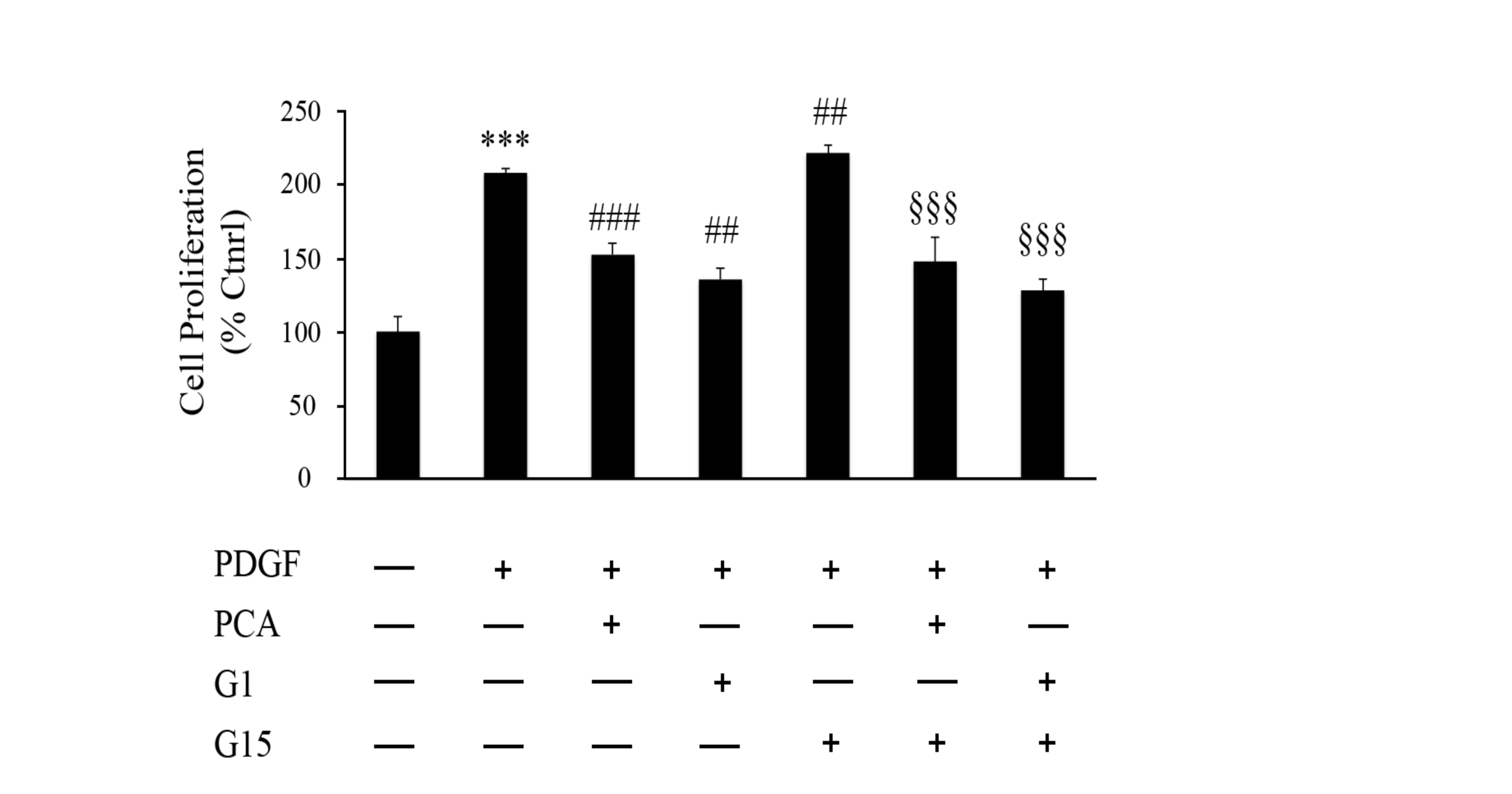

Supplement: Figure S1 — PCA's inhibition on VSMC cell proliferation. Media of VSMCs in 96 well were changed to serum free media for 24 hrs. Then, PCA (100 µM), G1 (3.0 µM) and G15 (3.0 µM) were treated for 24 hrs. After that, PDGF (10 ng/ml) was treated for 24 hrs. Graphs are representative of 3 independent experiments. *** indicates P<0.001 compared to the sham group. ##, ### indicates P<0.005, P<0.001, respectively to PDGF group. $$$ indicates P<0.0001 compared to PDGF+G15 group. Production will need this reference to link the reader to the figure. (TIF) [file pone.0113242.s001.tif]

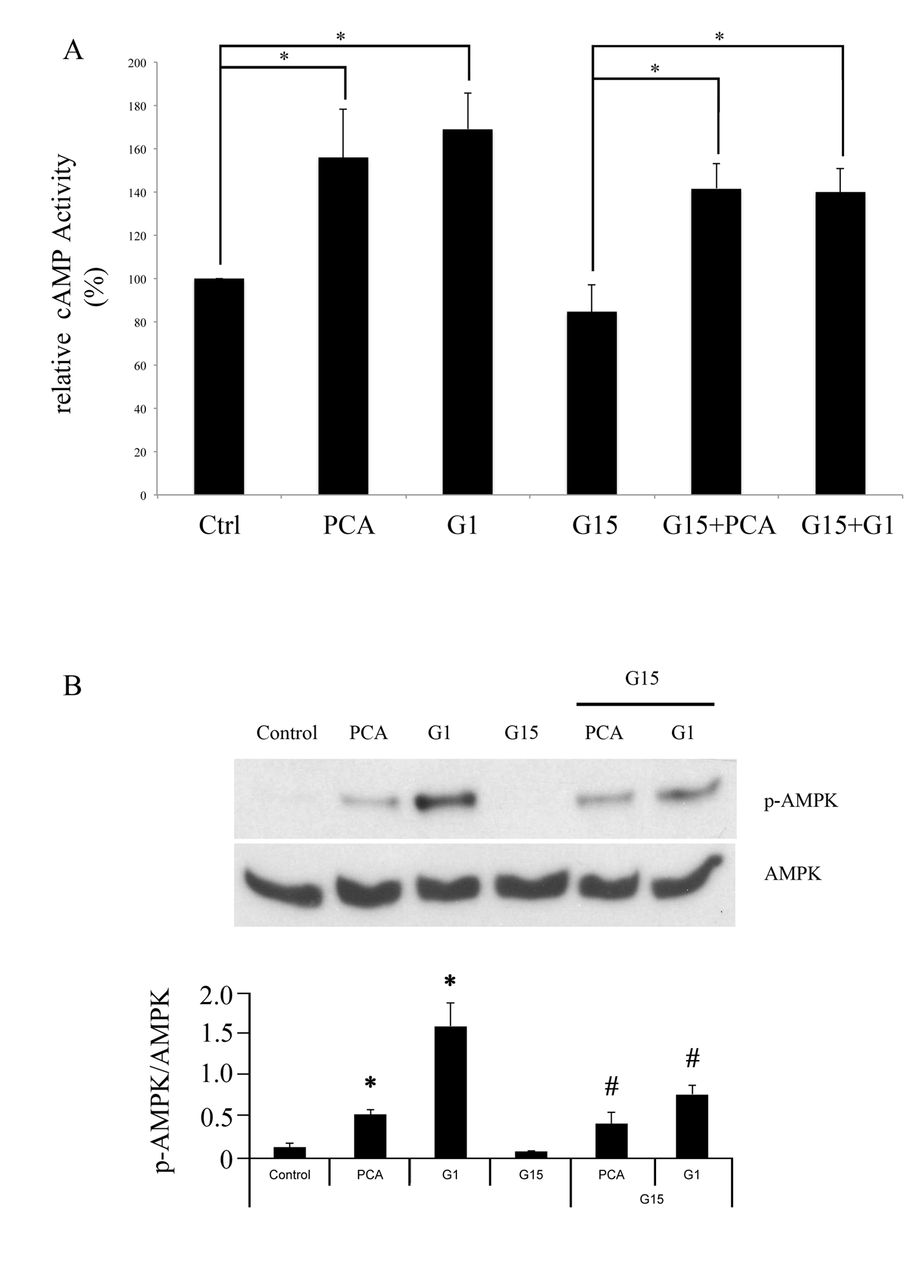

Supplement: Figure S2 — PCA and G15 effects on GPER-1 related mechanism. (A) HUVECs in 96 well were treated with adequate substances (PCA, G1, G15, G15+PCA, G15+G1) for 1 hr after 2 hrs of serum depletion. Graphs are representative of 3 independent experiments. * indicates P<0.05 compared to the control and G15 treated group. (B) HUVECs were pretreated with adequate substances for 24 hrs (PCA, 100 µM; G1, 3.0 µM). Then G15 (3.0 µM) were added for 6 hrs for p-AMPK and AMPK. Blots are representative of 3 independent experiments. * indicates P<0.05 compared to the control group. # indicates P<0.05 compared to the G15 group. (TIF) [file pone.0113242.s002.tif]
